# Advances in Triboelectric Nanogenerators for Microbial Disinfection

**DOI:** 10.3390/mi16030281

**Published:** 2025-02-27

**Authors:** Jinyoung Jeon, Donghyeon Kang, Sang-Woo Kim

**Affiliations:** 1 Department of Materials Science and Engineering, Yonsei University, Seoul 03722, Republic of Korea; 2 Center for Human-Oriented Triboelectric Energy Harvesting, Yonsei University, Seoul 03722, Republic of Korea

**Keywords:** triboelectric technology, microbial disinfection, electroporation

## Abstract

The global COVID-19 pandemic has highlighted the pivotal role of microbial disinfection technologies, driving the demand for innovative, efficient, and sustainable solutions. Triboelectric technology, known for efficiently converting ambient mechanical energy into electrical energy, has emerged as a promising candidate to address these needs. Self-powered electro-based microbial disinfection using triboelectric nanogenerators (TENGs) has emerged as a promising solution. TENGs have demonstrated effective disinfection capabilities in various settings, including water, air, surfaces, and wounds. This review explores the advancements in TENG-based microbial disinfection, highlighting its mechanisms and applications. By utilizing triboelectric technology, it provides comprehensive insights into the development of sustainable and efficient solutions for microbial control across diverse environments.

## 1. Introduction

Airborne pathogens spread rapidly, posing a significant threat to public health. In modern societies characterized by densely populated indoor environments, the risk of infection is heightened [1,2]. The COVID-19 pandemic, which claimed over 6 million lives globally, underscored the critical importance of pathogen prevention and control systems [3]. Consequently, the demand for effective microbial disinfection technologies has grown substantially. Traditional microbial disinfection methods, such as chemical disinfectants, high-temperature and high-pressure sterilization, and ultraviolet (UV) treatment, have been widely employed. However, these methods come with limitations. Chemical disinfectants may lead to secondary contamination and toxicity due to residual chemicals. And the emergence of chlorine-resistant pathogens has reduced the efficacy of chlorine-based disinfection methods. Hydrogen peroxide (H_2_O_2_), while effective, poses challenges in terms of transport and storage stability. High-temperature and high-pressure sterilization is energy-intensive and dependent on specialized equipment. UV disinfection requires prolonged exposure and may be less effective against certain pathogens or under specific environmental conditions [4,5,6,7]. To overcome these limitations, electro-based microbial disinfection technologies have gained increasing attention.

Electro-based disinfection utilizes electrical mechanisms to eliminate or inactivate pathogens [8]. This approach minimizes the use of chemicals, offers environmental friendliness, and enables rapid operation, making it an efficient and sustainable alternative to conventional disinfection methods. This approach encompasses three main mechanisms: electroporation, electrochemical processes, and electrostatic interactions. Electroporation uses strong electric fields to physically disrupt microbial cell membranes, leading to inactivation. Electrochemical processes generate reactive oxygen species (ROS), which oxidize and damage microbial internal structures. Electrostatic interactions leverage the forces between charged surfaces and pathogens to block or inactivate them. These technologies offer high energy efficiency and are adaptable to diverse environments, including water, air, and surfaces, making them a powerful alternative to conventional methods.

Triboelectric nanogenerators (TENGs) represent a promising energy source for electro-based disinfection technologies. TENGs convert mechanical energy into electrical energy and can harvest energy from various sources, such as body motion, wind, waves, and ultrasound [9,10,11,12]. Unlike traditional battery-based systems, which face limitations in size, lifespan, and waste generation, TENGs enable self-powered systems that operate without batteries. Additionally, TENGs can generate high voltages, making them suitable for producing electric fields or ROS for disinfection. TENG-based disinfection technologies can be applied effectively to water, air, surfaces, and even medical applications such as wound sterilization. Thus, TENG-driven electro-based microbial disinfection offers a sustainable solution that addresses both public health and environmental conservation [13]. This approach reduces chemical usage while maximizing pathogen control efficiency, unlocking innovative possibilities across various fields.

**Figure 1:**
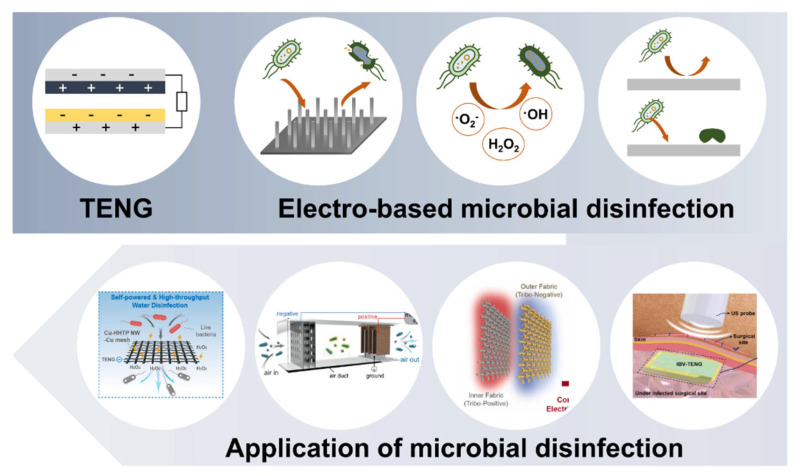
Schematic overview of TENG-driven electro-based microbial disinfection across various applications [14,15,16,17].

This review provides a comprehensive overview of microbial disinfection technologies based on triboelectric principles (Figure 1). First, the fundamental mechanisms of TENGs and advancements in triboelectric materials are examined to establish a technical foundation. Next, the review discusses electro-based microbial disinfection technologies, including electroporation, electrochemical processes, and electrostatic interactions, as applications of triboelectric technology. Furthermore, the performance and results of these technologies in water, air, surface, and wound disinfection are evaluated comprehensively. Finally, the challenges and prospects of triboelectric-based microbial disinfection are addressed, offering insights into future advancements in this field.

## 2. Working Principle and Materials of TENG

### 2.1. Working Mechanism of TENG

**Figure 2:**
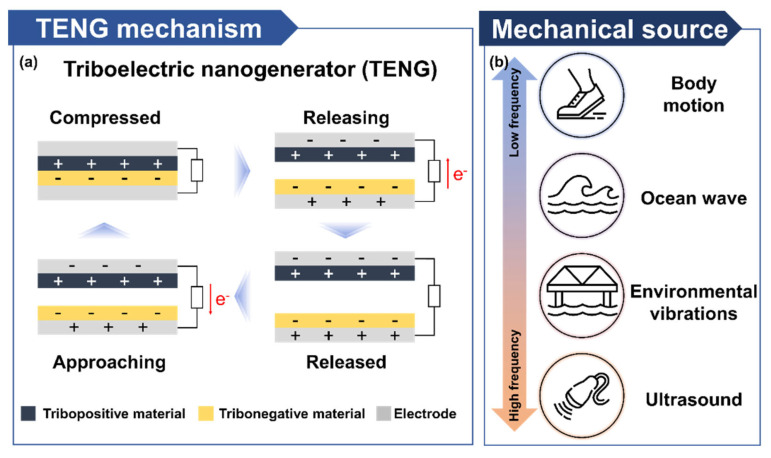
Comprehensive working mechanism of TENG. (**a**) Fundamental operating principle of a contact-separation mode TENG. (**b**) Various mechanical energy sources for driving TENG.

TENG has gained attention as a sustainable energy harvesting technology for self-powered electronic devices that operate without batteries. The overall working mechanism of TENG is illustrated in Figure 2a. It generates output by the coupling effect of contact electrification and electrostatic induction. When two different materials in the triboelectric layer come into contact and then separate, their surfaces become oppositely charged, a phenomenon determined by the materials’ tendencies to transfer electron [18]. This behavior is classified based on the Triboelectric Series, which categorizes materials according to their propensity to lose or gain electrons [19,20,21]. Materials classified as tribonegative have a strong tendency to gain electrons and acquire negative charges. In contrast, tribopositive materials tend to lose electrons and become positively charged after contact and separation. To maximize the efficiency of TENG, it is critical to pair materials with a significant electrical potential difference. The greater the difference in their triboelectric properties is, the more electrons are transferred during contact and separation, resulting in a stronger potential difference [22]. This potential drives the flow of electrons through an external circuit, generating an electric current. By optimizing the choice of tribonegative and tribopositive materials, TENG can achieve higher output and improved energy conversion efficiency.

This process of contact and separation is repeatedly driven by external mechanical forces. The mechanical energy required to operate TENG can come from diverse sources (Figure 2b), spanning a wide range of frequencies. These energy sources are classified into low-frequency and high-frequency mechanical energy. Low-frequency mechanical energy, such as that generated by body motion or organ movement, contrasts with high-frequency sources, including ultrasonic vibrations. TENG can also use mechanical energy from natural phenomena like wind and waves [10,11], as well as vibrations from artificial structures such as bridges [23,24]. Biomechanical energy, derived from human motion, is a readily accessible source for TENG operation. It enables repetitive activation through daily movements, making TENG particularly useful for self-powered devices such as wearable healthcare systems [25]. Ultrasound-based TENGs utilize the high-frequency, strong vibrations provided by ultrasound, which is not only efficient for power generation but also safe for biological applications [26,27,28]. As a result, TENGs are highly suited as energy sources for implant medical devices and other high-efficiency self-powered electronics.

TENG’s ability to produce high voltage and harvest energy across various environments makes it ideal for microbial disinfection technologies. TENGs can generate electric fields or induce reactive oxygen species (ROS) to effectively eliminate pathogens. These features position TENG as a sustainable energy solution with transformative potential across a wide range of applications.

### 2.2. Advanced Triboelectric Materials for High-Performance TENG

Extensive research has focused on developing high-performance TENGs with enhanced output and stable functionality, broadening their applicability across various fields. Key strategies for advancing TENG performance include incorporating high-dielectric nanoparticles, surface functionalization, micro/nanopatterned surface structure designs, and ultrasound-driven vibration control. Electroporation requires the formation of a sufficiently strong electric field exceeding 107 V m⁻¹ to induce irreversible microbial inactivation [29]. Additionally, in electrochemical disinfection, increasing the current density from 0.3 to 1 mA significantly reduced the inactivation time by half. Therefore, the development of high-performance TENGs is essential to enhance disinfection efficiency [30]. Table 1 summarizes TENGs with enhanced output achieved through various optimization strategies.

#### 2.2.1. High-Dielectric Composite

High-dielectric materials such as BaTiO_3_ and CaCu_3_Ti_4_O_12_ exhibit excellent charge trapping capabilities, significantly boosting TENG output. Surface charge density, a critical factor influencing TENG performance, can be enhanced by using dielectric materials with high charge-trapping capacities. Seung et al. synthesized a composite of BaTiO_3_ (BTO) and poly(vinylidenefluoride-co-trifluoroethylene) (P(VDF-TrFE)), achieving over 150 times higher output compared to Polytetrafluoroethylene (PTFE) [31]. Kelvin probe force microscopy (KPFM) analysis revealed a substantial increase in surface charge potential due to the ferroelectric polarization induced by the dielectric BTO nanoparticles within P(VDF-TrFE). With dielectric constants of 10.9 and 12.4 for polarized P(VDF-TrFE) and its BTO composite, respectively, the charge trapping capacity was maximized, resulting in an output of 1130 V and 1.5 mA.

Kim et al. developed a high-performance TENG using CaCu_3_Ti_4_O_12_ (CCTO) particles combined with a butylated melamine formaldehyde (BMF)-based composite [32]. CCTO particles demonstrated strong internal polarization under an electric field, enhancing charge induction and triboelectric output. This composite achieved a remarkable power output of 268 V and 25.8 mA/m^2^.

#### 2.2.2. Functional Group Modification

Kwak et al. enhanced TENG performance through the use of BMF, utilizing its highly positive triboelectric properties derived from abundant hydrogen atoms in its functional groups [33]. Hydrogen atoms donate electrons to the opposing material during friction, enabling BMF to form positive charges. Density functional theory (DFT) simulations confirmed that the outermost electron orbitals (HOMOs) of BMF contributed to its enhanced positive charge characteristics. BMF-based TENGs exhibited an output of 210 V and 125 μA, 2.3 and 4.2 times higher than PTFE-based TENGs, respectively.

#### 2.2.3. Micro/Nano-Patterned Surface

The micro/nano-patterned surface of the triboelectric layer typically enhances the TENG electrical performance by maximizing the deformation and contact area [35,36,37]. Bui et al. developed a TENG based on patterned polydimethylsiloxane (PDMS), achieving a relatively a high power density of 8.1 W/m^2^ with a very low applied force of 6.5 N [34]. Inspired by the micro-patterned structures of frog toe pads, they synthesized a similar convex-patterned PDMS and applied it to the triboelectric surface. By precisely controlling the pattern structure and spacing using a nonclose-packed microbead array, they achieved optimal deformation at a pattern spacing of 0.8 μm. The patterned triboelectric surface induced significant deformation in the contact area, effectively increasing the active triboelectric surface area and dramatically enhancing output performance. At a frequency of 5 Hz and an applied force of 38 N, the TENG demonstrated a power density of 23.9 W/m^2^, a voltage of 490 V, and a current density of 24.4 μA/cm^2^. This represented a sevenfold increase in power density compared to flat PDMS-based TENGs.

#### 2.2.4. Ultrasound Vibration Control

Chung et al. improved the power density of implant TENGs by 310% using liquid spaces within the packaging [38]. Conventional implant TENGs often use titanium (Ti) or its alloys for long-term sealing, but these materials reflect energy, reducing ultrasonic transmission efficiency. By controlling vibration modes, they achieved a power density of 3.85 mW/cm^2^ at 100 kΩ impedance, which was 310% higher than TENGs without Ti packaging and 6400% higher than Ti-packaged TENGs without liquid spaces. The incorporation of liquid spaces minimized air pockets, improved elastic wave transmission, and induced single-mode vibrations, significantly enhancing electrical output.

Kim et al. proposed using biocompatible 2-hydroxyethyl methacrylate (HEMA) as the encapsulation and triboelectric layer for ultrasound-driven TENGs [39]. Compared to Ti plates, HEMA-based packaging exhibited a 10-fold increase in ultrasonic transmission coefficients and charged a 100 μF capacitor 3.7 times faster. By using ethylene glycol dimethacrylate (EGDMA) as a crosslinker, the elasticity of HEMA was adjusted to match the impedance of surrounding tissues, optimizing performance under in vivo conditions. While the triboelectric output increased with crosslinker concentrations up to 3 wt%, it decreased at 5 wt% due to excessive stiffness and increased acoustic impedance, which hindered vibration transmission. This demonstrates the importance of matching the material’s acoustic impedance to human tissue for efficient power supply in biomedical applications.

## 3. TENG for Microbial Disinfection

**Figure 3:**
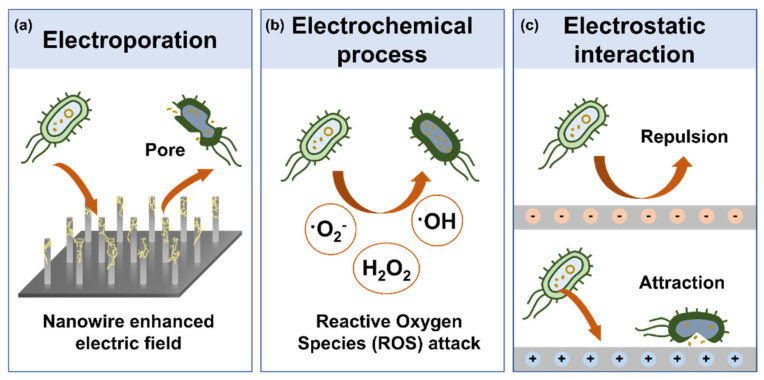
Mechanisms of electro-based microbial disinfection technologies. (**a**) Electroporation, which inactivates microbials by causing physical damage to their surfaces through concentrated electric fields. (**b**) Generation of ROS via electrochemical processes to inactivate microbials. (**c**) Microbial blocking and inactivation based on the electrostatic charging state of surfaces.

Disinfection, which involves the elimination or inactivation of microbials, plays a vital role in preventing infections across various fields, including healthcare, air purification, water management, and public health. Preventing the spread of pathogen-induced infections and developing disinfection methods that are safe for both the environment and human health are critical tasks for maintaining a sustainable public health system [40,41]. Electro-based microbial disinfection minimizes the use of chemical agents, thereby preventing secondary contamination. It also offers immediate disinfection effects and high efficiency. The mechanisms of electro-based microbial disinfection can be classified into electroporation, electrochemical processes, and electrostatic interactions (Figure 3).

### 3.1. Mechanism of Microbial Disinfection via TENG

Electroporation is a technique that utilizes electric fields to make nanometer-scale pores in microbial cell membranes. High-voltage electric pulses rearrange the lipid bilayer of cell membranes, inducing physical damage. Even after the removal of the electric pulses, strong electric fields cause irreversible damage to the microbial, ultimately leading to death. The disruption of the microbial membrane results in the breakdown of intracellular and extracellular material exchange, severely impairing critical life-sustaining activities such as energy metabolism. Irreversible pores in the membrane significantly increase permeability, preventing the microbial from performing essential functions and eventually causing death [42,43]. This mechanism, which physically disrupts the membrane without the use of chemical agents, has emerged as an environmentally safer and highly effective technology for microbial control. However, insufficient electric fields may create only temporary pores, allowing cells to repair themselves. Thus, optimizing the electric field strength and pulse conditions is critical for achieving irreversible microbial damage. High-aspect-ratio structures, such as nanowires and nanorods, are employed to generate high-voltage electric fields. These structures enhance local charge density at their tips, increasing electric field intensity and facilitating efficient electroporation. After electroporation, scanning electron microscopy (SEM) and transmission electron microscopy (TEM) can visually confirm the presence of pores in microbial [44]. Electroporation, being a physical and residue-free disinfection method, holds significant potential in applications such as microbial control, cell permeability modulation, drug delivery, and pathogen inactivation.

Electrochemical disinfection relies on ROS, which act as potent oxidants to inactivate microbials. ROS such as superoxide (O_2_⁻), hydroxyl radicals (•OH), and H_2_O_2_ disrupt microbial cell membranes and damage internal structures, leading to inactivation. ROS oxidize microbial proteins and DNA, impairing replication and transcription and thereby inhibiting normal cellular activities [45]. Additionally, chloride ions (Cl−) can participate in oxidation reactions to produce hypochlorous acid (HOCl), a compound that causes severe damage to microbials. Moreover, electrochemical disinfection can induce electron transfer within microbials, dramatically increasing intracellular ROS generation. This rapid ROS production creates oxidative stress, leading to critical cellular damage and disrupting metabolic pathways, providing a swift and effective mechanism for microbial inactivation [46]. TENGs can utilize their high-voltage output to generate ROS through electrochemical reactions to electrodes in water. By producing hydroxyl radicals (•OH) and hydrogen peroxide (H_2_O_2_), these reactions achieve powerful disinfection by oxidizing microbial protein structures and damaging cell membranes and energy systems.

Electrostatic interaction exploits the electrical attraction or repulsion between charged materials and pathogens to block or inactivate microbials. This approach operates by modulating surface charge properties and electrostatic forces, offering effective disinfection without chemical agents [47,48,49]. Most microbials possess a negative charge due to functional groups (e.g., carboxyl and phosphate groups) in their phospholipids, proteins, and polysaccharides located on cell membranes or capsids. These negatively charged pathogens interact with positively charged materials, forming electrostatic attractions. Such interactions can effectively trap and inactivate pathogens or damage their cell membranes, leading to the leakage of intracellular content and loss of cellular function. Conversely, negatively charged materials repel negatively charged pathogens, creating a barrier that prevents pathogen adhesion. This repulsion is particularly effective for airborne pathogen removal and is employed in personal protective equipment (e.g., masks and filters) and air purification systems to block pathogen penetration through filters. Electrostatic interaction-based disinfection technologies are promising for portable disinfection devices, personal protective equipment, and air purifiers. They offer environmentally friendly and energy-efficient solutions, presenting substantial potential in diverse disinfection applications.

### 3.2. Water Disinfection

Disinfecting microbials in water is essential for ensuring public health. Water serves as a primary medium for pathogen proliferation and transmission, and inadequate microbial control can lead to severe health and environmental issues. Conventional water disinfection methods, such as chlorine treatment, rely on extensive use of chemical agents, raising concerns about secondary contamination. In contrast, TENG offers a promising alternative for electro-based water disinfection due to its ability to generate electricity from mechanical energy and produce high-voltage output [50,51,52].

**Figure 4:**
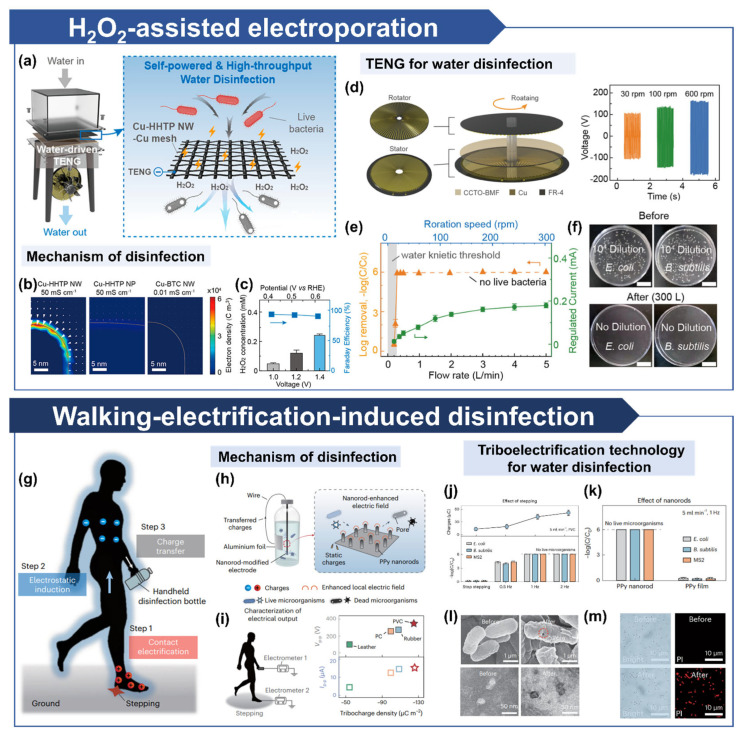
Electro-based microbial disinfection in water. H_2_O_2_-assisted electroporation for microbial disinfection in water: (**a**) Schematic representation of the overall H_2_O_2_-assisted electroporation mechanism. (**b**) Calculated electric field distribution of high-conductivity nanowires for electroporation. (**c**) H_2_O_2_ generation as a function of applied voltage using Cu-HHTP NW-Cu electrodes. (**d**) Schematic illustration of TENG for water disinfection and its output at various rpm. (**e**) TENG-based water disinfection efficacy as a function of flow rate and rotation speed. (**f**) Plating results of *E. coli* (left) and *B. subtilis* (right) before and after TENG disinfection (5 L/min, ~300 rpm) [14]. Walking-electrification-induced electroporated disinfection (WEED) system: (**g**) Schematic representation of the overall WEED system. (**h**) Mechanism of portable water disinfection using the WEED system. (**i**) Schematic image of electrical output measurement and comparison of triboelectric charge density based on shoe material. (**j**) Influence of step frequency on charge generation efficiency (top) and microbial disinfection effectiveness (bottom). (**k**) Disinfection efficiency based on electrode shape for electroporation. (**l**) SEM and TEM images of bacteria and viruses before and after disinfection. (**m**) Bright-field (total bacteria) and fluorescence (inactivated bacteria) microscopy images of *E. coli* before and after disinfection [53].

Huo et al. proposed a hydrogen peroxide-assisted electroporation-based water disinfection system powered by a flow-driven TENG (Figure 4a) [14]. This system employs a direct current (DC)-based TENG coupled with tricatecholate (2,3,6,7,10,11-hexahydroxytriphenylene) with copper(II) ions (Cu-HHTP) nanowire (NW)-Cu nanowire electrodes integrated into a disinfection filter. As bacteria-laden water passes through the filter, disinfection occurs via local electric fields and hydrogen peroxide generation facilitated by the nanowires. High-aspect-ratio structures enhance local electric fields by concentrating free charges at regions with the smallest curvature radius, whereas nanoparticles (NPs) exhibit insufficient charge accumulation and fail to create strong electric fields (Figure 4b). Additionally, even among NW structures, Cu-HHTP NW (~50 mS/cm) demonstrated superior field enhancement compared to Cu-benzene-1,3,5-tricarboxylate (Cu-BTC) NW (<10^−2^ mS/cm), which has limited conductivity. The excellent disinfection performance (>6.0-log) in river and tap water verified its potential for actual applications. Conventional TENG-driven electroporation methods were unable to achieve immediate inactivation under increased throughput. However, the combination of electroporation with H_2_O_2_ significantly enhanced the disinfection performance, enabling complete microbial inactivation even at higher throughput levels. These findings highlight the effectiveness of high-conductivity, high-aspect-ratio Cu-HHTP NWs in enabling efficient electroporation.

Moreover, the system generates hydrogen peroxide in high concentrations without chemical consumption by utilizing electrochemical reduction of dissolved H_2_O_2_ at low driving voltages (Figure 4c). The TENG employed consists of a rotary design with Cu electrodes and a stator coated with CCTO-particle-doped BMF. High durability and triboelectric properties of BMF, combined with the high dielectric constant of CCTO, enabled high output power (Figure 4d). This system achieved complete bacterial inactivation over a wide range of flow rates and maintained disinfection efficiency at low flow rates and rotational speeds, demonstrating significant potential for practical applications (Figure 4e,f). It highlights a system that synergistically combines electroporation and electrochemical processes, achieving enhanced throughput and disinfection efficiency.

Kim et al. introduced a field-disinfection technology using triboelectric charges generated through walking, offering a practical solution for providing clean water in rural and disaster-affected areas (Figure 4g) [53]. Triboelectric charges generated during daily activities induce charges on the surface of a portable disinfection bottle via contact electrification and electrostatic induction. These charges contribute to electroporation by forming localized electric fields around nanorod tips, leading to microbial inactivation (Figure 4h). Analysis of triboelectric charges generated by different shoe materials confirmed that poly(vinyl chloride) (PVC) produced sufficient charge density for effective disinfection (Figure 4i). Disinfection efficiency driven by walking-induced electrostatic charges was further examined, demonstrating complete microbial inactivation at a step rate of 1 Hz, corresponding to an electrostatic charge exceeding 42 nC (Figure 4j). Further studies demonstrated that only nanorods enabled successful microbial disinfection, as flat surfaces failed to generate adequate electric fields (Figure 4k). The effectiveness of the technique was validated by SEM and TEM imaging, which revealed pore formation on Escherichia coli (*E. coli*) and bacteriophage MS2 membranes (Figure 4l). More than 95% of the bacterial cells exhibited red fluorescence after treatment, stained with propidium iodide, indicating enhanced membrane permeability due to electroporation (Figure 4m). This approach demonstrates a portable, field-based disinfection solution that eliminates the need for centralized water treatment systems by utilizing triboelectric charges.

### 3.3. Air Disinfection

Pathogens spreading through the air in densely populated spaces pose significant threats. Pathogenic microbials contained in aerosols are easily released into the air, traveling several kilometers and increasing the risk of infection [54,55]. Conventional high-efficiency particulate air (HEPA) filtration, while widely used, has limitations, including pressure drops, inability to inactivate microbials, and the need for frequent filter replacements. Additionally, employing electroporation for microbial disinfection in indoor environments requires overcoming challenges associated with rapid airflow in ventilation systems.

**Figure 5:**
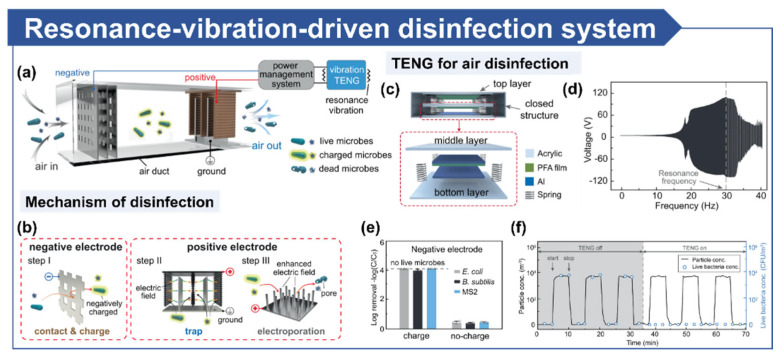
Electro-based microbial disinfection in air. (**a**) Schematic of the resonance-vibration-driven disinfection system. (**b**) Overall microbial disinfection mechanism involving microbial charging, trapping, and electroporation. (**c**) Structure of the resonance-vibration-driven TENG. (**d**) Output variation of TENG with respect to frequency. (**e**) Disinfection performance based on negative charge electrode. (**f**) Changes in microbial concentration using bacterial bioaerosols according to TENG operation [15].

Kim et al. proposed a high-efficiency microbial disinfection system using a vibration-driven TENG (V-TENG) to address the limitations of rapid ventilation airflow (Figure 5a) [15]. The disinfection process consists of two primary stages (Figure 5b). In the first stage, air containing microbials passes through a porous cathode, which induces a negative charge on the microbials. In the second stage, the microbials are trapped on the surface of the positively charged anode under a strong electric field generated between the anode and a grounded electrode. Subsequently, the microbials are inactivated via electroporation facilitated by nanowires. The V-TENG acts as the power source for air disinfection. The TENG is composed of a spring structure with three layers (Figure 5c). Vibrations from a ventilator induce repeated contact and separation, generating power. The system achieved maximum output at a resonance frequency of 30 Hz, which aligns with the frequency of typical ventilation systems (Figure 5d). Experimental results demonstrated that bacteria surviving in aerosols were undetectable only when the TENG was intermittently activated, confirming its disinfection performance. This highlights the critical role of microbial charging in enhancing disinfection performance (Figure 5e,f). Electroporation relies on localized electric fields concentrated at the tips of nanowires, making it essential for microbials to reach these regions. Under high airflow conditions, charged microbials exhibited significantly higher inactivation efficiency compared to uncharged microbials. This study emphasizes the effectiveness of a V-TENG operating at resonance frequencies similar to those of building ventilation systems, enabling efficient electroporation-based air disinfection even in rapid airflow environments.

### 3.4. Surface Disinfection

Airborne pathogens are pervasive and can spread rapidly, exponentially increasing the risk of infection. To prevent pathogen transmission, the importance of personal protective equipment has become increasingly apparent. For devices like masks, which protect individuals from airborne microbials, surface disinfection plays a critical role [56,57]. While antimicrobial fabrics are effective at blocking microbials, they often come with high costs and the potential toxicity of embedded metals.

**Figure 6:**
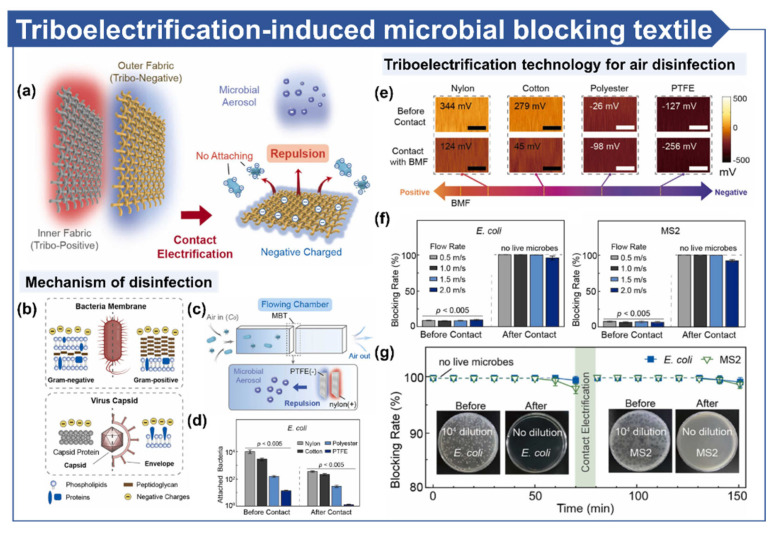
Electro-based microbial disinfection on surfaces. (**a**) Schematic illustration of the triboelectrification-induced microbial blocking textile. (**b**) Schematic depiction of the negatively charged surfaces of bacteria and viruses. (**c**) System setup of the microbial blocking textile installed within an air duct. (**d**) Microbial blocking efficiency before and after contact using various outer fabrics (**e**) Differences in surface potential induced by triboelectric charging of various materials. (**f**) Blocking efficiency of bacteria and viruses before and after contact at different airflow rates. (**g**) Long-term operational performance [16].

Suh et al. developed a microbial-blocking and infection-prevention technology based on electrostatic repulsion between fabrics and microbials (Figure 6a) [16]. Microbials (bacteria and viruses) in aerosols carry a negative charge, enabling them to be effectively repelled by similarly charged surfaces. The study emphasized that such charged surfaces can be easily generated using triboelectric charges produced during human motion. Aerosol-contained bacteria and viruses exhibit negatively charged surface potentials derived from their phospholipid membranes and capsids (Figure 6b). When microbial-laden air flows through PTFE and nylon fabrics (Figure 6c), these microbials are effectively blocked. The microbial blocking efficiency improved as the negative potential of various textiles increased. After BMF electrification, the number of *E. coli* detected on nylon, cotton, polyester, and PTFE surfaces significantly decreased from 9.4 × 10^3^, 2.1 × 10^3^, 1.2 × 10^2^, and 10 to 1.1 × 10^3^, 6.0 × 10^2^, 56, and 2, respectively (Figure 6d). PTFE and nylon were selected as materials for their highly negative and positive triboelectric properties, respectively (Figure 6e). As a result, the triboelectrification-induced microbial blocking textile (TE-MBT) system achieved complete microbial blocking even under high airflow speeds of 1.5 m/s, but only when triboelectric induction occurred (Figure 6f). Moreover, it demonstrated continuous microbial blocking via the regeneration of triboelectric charges (Figure 6g). This research highlights a promising method for microbial blocking to prevent infections, emphasizing its potential application in public health for personal infection prevention.

### 3.5. Antibacterial Wound Care

Prevention of surgical site infections (SSIs) is critical for reducing postoperative complications and ensuring patient recovery. SSIs increase postoperative mortality 11-fold and result in an economic burden exceeding USD 3 billion annually, while imposing significant physical and financial stress on patients [58,59]. Traditional infection prevention methods using antibiotics face limitations due to the emergence of antibiotic resistance and mutated microbials, underscoring the growing need for non-drug-based antimicrobial technologies.

**Figure 7:**
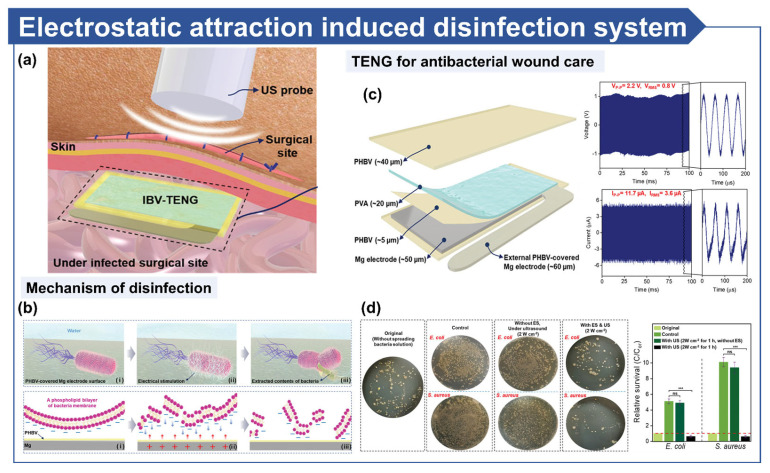
Electro-based microbial disinfection in wounds. (**a**) Schematic illustration of TENG-driven microbial disinfection of surgical site. (**b**) Mechanism of microbial inactivation through electrostatic attraction (i: a bacterium is approaching the surface before attachment. ii: electrostatic attraction induces electrical stimulation, iii: the bacterial membrane collapses, leading to leakage of intracellular components and inactivation). (**c**) Structure of an implantable, biodegradable, and vibrant TENG and its output under 20 kHz frequency and 2 W cm^−2^ ultrasonic power. (**d**) Bacterial disinfection efficiency under ex vivo conditions with TENG and ultrasound (“ns” means no significant difference and values of *** *p* < 0.001 are considered statistically significant) [17].

Imani et al. introduced implantable, biodegradable, and vibrant TENG (IBV-TENG), a biodegradable TENG system designed to inactivate microbials at surgical sites and degrade post-use without requiring additional surgical procedures (Figure 7a) [17]. IBV-TENG generates charges that induce attractive forces between negatively charged microbials and electrodes, forming a potential difference. This disrupts electron transfer within microbials and alters the polarity of their membrane surfaces, leading to structural destruction (Figure 7b). The IBV-TENG system employs Poly (3-hydroxybutyric acid-co-3-hydroxyvaleric acid) (PHBV) as the tribopositive material and Poly(vinyl alcohol) (PVA) as the tribonegative material to generate electrical output through ultrasound vibrations (Figure 7c). Experiments involving bacterial solutions on porcine tissue demonstrated that only IBV-TENG systems with electrical stimulation successfully inactivated microbials (Figure 7d). The system’s biodegradability and biocompatibility enable infection control at surgical sites while eliminating the need for secondary surgical removal. This research highlights the potential of TENG technology not only to prevent SSIs but also to minimize patient discomfort through its biodegradable properties, paving the way for innovative and patient-centric solutions in infection control.

## 4. Challenges and Perspective

TENG-based microbial disinfection systems are gaining attention as innovative alternatives that overcome the limitations of conventional methods, such as chemical residues and high energy consumption. These systems have the potential to establish themselves as sustainable technologies that replace or complement existing disinfection methods in public health, medical, and environmental protection applications. TENGs provide the electrical and physical mechanisms required for pathogen disinfection through self-powered energy generation and high-voltage output [60,61,62,63]. Their adaptability to diverse environments and conditions highlights their broad application potential. While TENG-based systems present a promising eco-friendly solution to microbial disinfection, several challenges remain. These include the development of durable TENGs capable of stable operation in various environments, achieving high-efficiency microbial disinfection, expanding applications to in vivo microbial control, and integrating monitoring systems. Addressing these challenges could enable TENG-based disinfection systems to evolve into practical solutions with significant contributions across multiple fields.

### 4.1. Development of Durable TENG

To ensure consistent and high output, maintaining output stability is essential. Particularly, for stable TENG operation under diverse environmental conditions, the durability and physical properties of triboelectric materials play a critical role. The long-term reliability of these triboelectric materials directly determines the efficiency and practical applicability of TENGs. The repetitive contact and friction experienced by the triboelectric layer can lead to physical wear and material degradation, compromising long-term stability. Addressing these issues requires the development of highly durable triboelectric materials and advanced surface design technologies. Recently, self-healing triboelectric materials have been employed to improve TENG durability [64]. Sun et al. developed a self-healing TENG using a linear silicone-modified polyurethane (PU) coating as the triboelectric layer [65]. Abrasion-induced damage was repaired through hydrogen bonding, completing the self-healing process within 30 min at room temperature without external solvents or agents. Notably, the device retained its performance post-healing, advancing TENG durability research. Additionally, selecting materials suited to specific environmental conditions is crucial for ensuring stable TENG performance. Applying materials resistant to external factors such as humidity, temperature, and pH fluctuations enables consistent output and reliability over extended periods. These material and design strategies not only enhance the durability and efficiency of TENGs but also ensure reliable performance across various applications.

### 4.2. High-Efficiency Microbial Disinfection

Achieving high-efficiency microbial disinfection requires maximizing TENG output, ROS generation, and the charge efficiency of the contact layer. This can be accomplished through the optimization of individual technologies as well as the integration of multiple disinfection mechanisms into hybrid systems. For instance, a hybrid system combining TENG and piezoelectric technology can leverage the complementary electrical output characteristics of both, providing a high voltage and stable energy supply [66]. This approach significantly enhances microbial disinfection efficiency. Such comprehensive strategies enable disinfection solutions capable of achieving performance levels that are difficult to attain with single technologies, ensuring robust operation under diverse environmental conditions. These advances not only enhance disinfection performance but also pave the way for the development of commercially viable microbial disinfection systems applicable across a wide range of practical applications.

### 4.3. Expanding Applications to In Vivo

Electro-based microbial disinfection technologies have demonstrated significant potential in applications involving air, water, and wearable devices, but their research and application in in vivo settings remain relatively underexplored. Infections within the body are among the leading causes of postoperative complications. Without effective microbial control, such infections can escalate to severe conditions such as sepsis, SSIs, and device-associated infections. Electro-based in vivo microbial disinfection technologies offer a powerful alternative to traditional antimicrobial approaches by overcoming their limitations. These technologies enable the localized application of electric fields, minimizing the impact on surrounding healthy tissues while effectively eliminating pathogens. Moreover, incorporating biodegradable triboelectric materials eliminates the need for additional removal surgeries after in vivo use, thereby reducing the patient’s physical burden and infection risks. These advancements pave the way for a new paradigm in in vivo microbial disinfection, offering practical solutions in diverse medical fields, including infection prevention in medical devices, chronic wound management, and SSI control.

### 4.4. Integrating Monitoring Systems

The integration of real-time monitoring and feedback systems further enhances disinfection processes by optimizing costs and maximizing efficiency. Continuous monitoring of ROS generation and charge states enables automatic adjustments to counteract any imbalances during the disinfection process. This ensures adequate stimulation for pathogen removal while minimizing potential side effects on surrounding environments. By improving disinfection efficiency and reducing energy consumption, such systems enhance the long-term stability and cost-effectiveness of the technology. The adoption of real-time feedback systems will be a pivotal step in advancing next-generation electro-based disinfection technologies into safe and reliable solutions.

## Figures and Tables

**Table 1:** Comparison of TENG performance by material or structure.

Materials/Structure	Performance	Reference
Non poled poly(vinylidenefluoride-co-trifluoroethylene) (P(VDF-TrFE))	30 V	[31]
Poled P(VDF-TrFE):BaTiO_3_ (BTO)	1130 V	[31]
Butylated melamine formaldehyde (BMF):CaCu_3_Ti_4_O₁_2_ (CCTO)	268 V	[32]
Polytetrafluoroethylene (PTFE)	90 V	[33]
BMF	210 V	[33]
Flat polydimethylsiloxane (PDMS)	200 V	[34]
Micropatterned PDMS	490 V	[34]
